# Association of genetic variants of oxidative stress responsive kinase 1 (*OXSR1*) with asthma exacerbations in non-smoking asthmatics

**DOI:** 10.1186/s12890-021-01741-x

**Published:** 2022-01-04

**Authors:** Min-Hye Kim, Hun Soo Chang, Jong-Uk Lee, Ji-Su Shim, Jong-Sook Park, Young-Joo Cho, Choon-Sik Park

**Affiliations:** 1 Department of Internal Medicine, College of Medicine, Ewha Womans University, Seoul, South Korea; 2 Department of Anatomy and BK21 FOUR Project, College of Medicine, Soonchunhyang University, Cheonan, South Korea; 3 Division of Allergy and Respiratory Medicine, Department of Internal Medicine, Soonchunhyang University Bucheon Hospital, 1174, Jung-Dong, Wonmi-Ku, Bucheon, Gyeonggi-Do 420-020 South Korea

**Keywords:** Asthma, Polymorphism, Exacerbation, Non-smokers

## Abstract

**Background:**

Asthma exacerbation threatens patient's life. Several genetic studies have been conducted to determine the risk factors for asthma exacerbation, but this information is still lacking. We aimed to determine whether genetic variants of Oxidative Stress Responsive Kinase 1 (*OXSR1*), a gene with functions of salt transport, immune response, and oxidative stress, are associated with exacerbation of asthma.

**Methods:**

Clinical data were obtained from 1454 asthmatics and single nucleotide polymorphisms (SNPs) of *OXSR1* were genotyped. Genetic associations with annual exacerbation rate were analyzed depending on smoking status.

**Results:**

Eleven SNPs were selected using Asian data in the International HapMap database. The common allele of *rs1384006* C > T of *OXSR1* showed a significantly higher annual exacerbation rate than the rare allele in non-smoking asthmatics (CC vs. CT vs. TT: 0.43 ± 0.04 vs. 0.28 ± 0.03 vs. 0.31 ± 0.09, *P* = 0.004, *P*corr = 0.039). The frequent exacerbators had a significantly higher frequency of the common allele of *rs1384006* C > T than did the infrequent exacerbators (74.4% vs. 55.2%, *P* = 0.004, *P*corr = 0.038).

**Conclusion:**

The common allele of *rs1384006* C > T of *OXSR1* was associated with the asthma exacerbation rate and a higher risk of being a frequent exacerbator, indicating that non-smoking asthmatics who carry common alleles may be vulnerable to asthma exacerbations.

**Supplementary Information:**

The online version contains supplementary material available at 10.1186/s12890-021-01741-x.

## Introduction

Asthma is a heterogeneous disease of chronic airway obstruction with a wide variety of symptoms, which develops in response to genetic and environmental influences [1, 2]. Recent cluster analyses have demonstrated an exacerbation-prone phenotype in a certain number of asthmatics [3, 4]. Because asthma exacerbation is a potentially life-threatening condition, risk factors for exacerbation-prone asthma have been under intense research to assist in early diagnosis and the development of new treatment strategies. During the past decade, hypothesis-driven and hypothesis-free approaches about genetic factors and gene-environment interactions have been applied and many possibly associated genetic variants have been identified, including several single nucleotide polymorphisms (SNPs) [1, 2]. For example, a mutant allele of *rs1800925* on *IL13* was associated with emergency room (ER) visits or hospitalizations of Costa Rican children with asthma [5], and those of *rs1805011* and *rs1801275* on *IL4RA* were associated with intensive care unit (ICU) care, ER visits or hospitalizations in two cohorts of US adult asthma patients [6]. The mutant allele of *rs4950928* on *CHI3L1* was also associated with asthma-related hospital admissions in adult and pediatric asthmatics [7]. The SNP *rs7216389* of *ORMDL3* was associated with exacerbation of asthma of children between the ages of 1–6 years [8]. Variants of *CD14* SNP *rs2915863* and *LY96* SNP *rs17226566* were also related to the risk of acute severe exacerbations induced by environmental endotoxin exposure [9]. However, additional genetic factors associated with asthma exacerbation should be elucidated for in-depth understanding of the genetic pathogenesis and the improvement of diagnostic accuracy.

Patients with severe asthma, including exacerbation-prone asthma, have current unmet needs in terms of a lack of effective treatments, such as corticosteroids [10]. Corticosteroid insensitivity is a clinical feature of severe asthma and COPD, as characterized by the reduced effect of dexamethasone in inhibiting the release of proinflammatory cytokines from monocytes and macrophages [11]. Activation of p38 mitogen activated protein kinase (MAPK) may alter corticosteroid responsiveness in response to oxidative stress and enhanced oxidative stress is one of the main triggers inducing chronic airway inflammation [12]. Excessive generation of reactive oxygen species (ROS) has been shown to activate multiple protein kinases, such as extracellular signal-regulated kinase (ERK)1/2, protein kinase B (PKB), and protein tyrosine kinases (PTKs) [13, 14]. Oxidants-induced mucin production from epithelial cells was accompanied by p38 MAPK activation resulting from a decrease in function of the tyrosine phosphatase Src homology region 2 domain-containing phosphatase-1 (SHP-1) [15].

One of the important clinical manifestations of exacerbation is an increase in the production of and alterations of the nature of mucus. A recent quantitative pathology analysis of fatal asthmatics found that more than 98% of their airways were occluded to some extent by mucus [16]. In addition, acute exacerbation with airway obstruction is usually caused by a mucus plug in the large and medium-sized bronchi, and even in the small airways [17]. The nature of mucus is regulated by the dilution of bronchial epithelial lining fluids. The transport of anions such as Cl– and HCO_3_– in the airway epithelium is recognized as one of the most important factors to regulate airway surface hydration and mucociliary clearance [18]. ROS induces lipid peroxidation of cell membranes and the oxidation of amino acids to inactivate membrane-bound receptors [19, 20]. This damage may modify the functions of membrane molecules, such as cystic fibrosis transmembrane conductance regulator (CFTR) and solute carrier family 26 member 4 (Slc26a4) [21].

The WNK-SPAK/OXSR1 kinase complex is composed of the kinases WNK (with no lysine) and SPAK (SPS1-related proline/alanine-rich kinase) or the SPAK homolog OXSR1 (oxidative stress–responsive kinase 1). The WNK family senses changes in intracellular Cl^−^ concentration, extracellular osmolarity, and cell volume and transduces this information to Na^+^, K^+^, and Cl^−^ cotransporters (collectively referred to as CCCs [cation-chloride cotransporters)] and ion channels to maintain cellular and organismal homeostasis. WNK1 phosphorylates and activates two related kinases, OXSR1 and STK39, which in turn phosphorylate and activate the Na^+^-K^+^-Cl^−^ co-transporters: SLC12A2 (NKCC1) and SLC12A1, SLC26A3, SLC26A6, SLC26A9 [22], CFTR [23], and the Cl^−^ and/or HCO_3_^−^ transporters NBCe1-B [24, 25].

*OXSR1* is ubiquitously expressed in most tissues, with high levels in the lung, especially the bronchial epithelium (https://www.proteinatlas.org/). In addition, *OXSR1* is also thought to play an important role in regulation of immune response and oxidative stress [26, 27]. These data prompted us to study the association of genetic variants of *OXSR1* with the risk of asthma exacerbation.

## Materials and methods

### Study subjects

The study subjects were Korean asthma patients who met the following diagnostic criteria: physician-diagnosed asthma with airway reversibility (more than 12% and 200 mL increase in forced expiratory volume in one second (FEV1), more than 20% change in peak expiratory flow rate), 20% or more of FEV1 improvement after asthma treatment for 2 weeks, or provocative concentration 20 (PC20) < 10 mg/mL on methacholine bronchial provocation test. They were followed up to for longer than 1 year after enrollment at 3 tertiary hospitals.

DNA from 1454 asthmatic patients who met these conditions were obtained from the biobank of Soonchunhyang University Bucheon Hospital, Korea and written informed consent was obtained at the time of DNA collection. The number of exacerbations was measured for the initial 1 year after enrollment, and asthma exacerbation was defined as the definition used by the American Thoracic Society/European Respiratory Society, which includes both severe and moderate exacerbation [28]. Severe exacerbation was defined as a condition that needs the addition of systemic corticosteroids (> 0.5 mg of prednisolone/kg of body weight for more than 3 days) and consideration of a hospitalization or emergency room (ER) visit, and moderate exacerbation was defined as an exacerbation that is improved by increasing other asthma medications, such as inhaled corticosteroids (ICS), or by adding a rescue bronchodilator without using systemic corticosteroids [28]. Pulmonary function was measured at baseline and then every three months, and the ICS and systemic corticosteroids used were expressed as fluticasone equivalent dose (mcg/day) and prednisone equivalent dose (mg/year) as previously described [29]. The protocol was approved by the Ethics Committee of Soonchunhyang Bucheon Hospital (SCHBC_2014_07_028).

### Selection of single nucleotide polymorphisms and genotyping

Single nucleotide polymorphisms (SNPs) in *OXSR1* were selected using the Asian population database from the International HapMap Project (http://hapmap.ncbi.nlm.nih.gov/) and NCBI (http://www.ncbi.nlm.nih.gov/snp) databases as follows: first, candidate SNPs were extracted from the intragenic region including 2 kb of the 5’ region of the gene using Asian population data in the International HapMap database, and then linkage disequilibrium structures of each gene were analyzed using SNPs with > 5% minor allele frequencies (MAF). A representative SNP was selected in the case of absolute LD (|D′| = 1 and r2 > 0.95) between the SNPs. Finally, 11 SNPs were selected and genotyped using the GoldenGate assay with VeraCode microbeads (Illumina, San Diego, CA, USA) as previously described [30]. These were scanned using the BeadXpress® system (Illumina).

### Statistics

Fisher’s exact test was used to compare the observed number of each genotype with those expected for a population in Hardy–Weinberg equilibrium (HWE). Haplotypes of each individual were inferred using the PHASE algorithm (ver. 2.1) [31]. A type III univariate general linear model was applied to the continuous variables (number of exacerbations) and multiple logistic regression to the discrete variables (presence of frequent exacerbation). In the logistic regression analysis, the odds ratios (ORs) and 95% confidence interval were calculated for each genotype and haplotype. The data were analyzed using SAS ver. 9.1 (SAS, Cary, NC, USA) and SPSS ver. 12.0 (SPSS, Chicago, IL, USA). To correct the P-values for multiple comparisons, the effective number of independent SNPs (M_eff_) of *OXSR1* was calculated using SNP spectral decomposition (http://genepi.qimr.edu.au/general/daleN/SNPSpD) [32]. The calculated M_eff_ value for the 11 SNPs of *OXSR1* was 10.035. Corrected *P* (*P*corr) values < 0.05 were considered significant. Statistical power of the genetic association was calculated using the Genetic Association Study (GAS) Power Calculator (http://csg.sph.umich.edu/abecasis/cats/gas_power_calculator/) based on CaTS power calculator [33].

## Results

### Clinical characteristics of the study subjects

A total of 1,454 asthma patients were enrolled (Table 1). Their clinical characteristics were compared depending on their exacerbation frequency: frequent exacerbators (FE) were defined as subjects experiencing exacerbations 2 or more times in the first year of follow-up.

**Table 1:** Clinical characteristics of the study subjects

	Infrequent exacerbator	Frequent exacerbator*	*P*
Number	1328	126	–
Age (years)	46.27 ± 0.43	51.2 ± 1.16	1.05E−04
Sex (male %)	38.10%	41.30%	0.503
Number of exacerbation in the first year	0.25 ± 0.02	2.03 ± 0.11	1.21E−32
Smoking status (non-smokers/ex-smokers/smokers, %)	66.3/18.5/15.1%	58.7%/22.2%/19.1%	0.086
Smoking amount (pack-year)	5.86 ± 0.36	8.47 ± 1.46	0.084
Atopy (%)	48.80%	39.70%	0.05
Duration of asthma (years)	3.06 ± 0.2	5.95 ± 0.94	0.003
Duration of follow-up (years)	6.17 ± 0.12	7.29 ± 0.37	0.007
Serum total IgE (IU/ml)	356.3 ± 18.87	453.17 ± 62.05	0.131
Body mass index (kg/m^2^)	23.87 ± 0.12	23.96 ± 0.47	0.859
Baseline FVC%, predicted	83.02 ± 0.48	67.65 ± 1.4	1.45E−20
Baseline FEV1%, predicted	82.58 ± 0.6	58.4 ± 1.59	3.06E−30
Baseline FEV1/FVC	75.64 ± 0.34	64.81 ± 1.15	6.96E−20
PC20, methacholine (mg/ml) (No. of study subjects)	7.6 ± 0.29 (1,218)	5.52 ± 0.83 (109)	0.037
Total ICS dosage used in the 1st year (Fluticasone eqv./day)	211.36 ± 7.25	571.34 ± 43.82	3.16E−13
Systemic prednisolone dose in the 1st year (mg/year)	80.31 ± 9.55	564.14 ± 95.39	1.51E−06

Data are expressed as mean ± standard error

*Frequent exacerbator is defined as a subject experiencing an exacerbation 2 or more times in the first year of follow-up

When compared with non-frequent exacerbators (non-FE), the FE had an older age (51.2 ± 1.2 vs. 46.3 ± 0.4 years, *P* = 1.05E−04), higher smoking amount (8.5 ± 1.5 vs. 5.9 ± 0.4 pack year, *P* = 0.041), and lower rate of atopy (39.7 vs. 48.8%, *P* = 0.05). Durations of asthma and follow-up were longer in the FE (6.0 ± 0.9 vs. 3.1 ± 0.2 years, *P* = 0.003, 7.3 ± 0. vs. 6.2 ± 0.1 years, respectively, *P* = 0.007).

In the FE group, forced vital capacity (FVC), FEV1, and FEV1/FVC were all significantly lower (67.7 ± 1.4vs. 83.0 ± 0.5% predicted, *P* = 1.45E−20, 58.4 ± 1.6vs. 82.6 ± 0.6% predicted, *P* = 3.06E−30, 64.8 ± 1.2vs. 75.6 ± 0.3%, respectively, *P* = 6.96E−20), and the PC20 of the methacholine challenge test was also lower (5.5 ± 0.8 mg/mL vs. 7.6 ± 0.3 mg/mL, *P* = 0.037) than those of the non-FE.

Doses of ICS and systemic corticosteroids used for the first year were higher in the FE (571.3 ± 43.8 vs. 211.4 ± 7.3 μg/day of fluticasone equivalents, *P* = 3.16E−13, 564.1 ± 95.4 mg/year vs. 80.3 ± 9.6 mg/year of prednisolone equivalents, *P* = 1.51E−06). Asthma medications except glucocorticosteroids of the study subjects were presented in Additional file 1: Table S1. Age, sex, serum total IgE level, predicted FEV1% at the first visit, and total ICS and systemic steroid dose in the 1st year of visit were considered covariates in the analyses of genetic associations.

### Frequencies, heterozygosity, and Hardy–Weinberg equilibrium (HWE) of SNPs of OXSR1

**Fig. 1:**
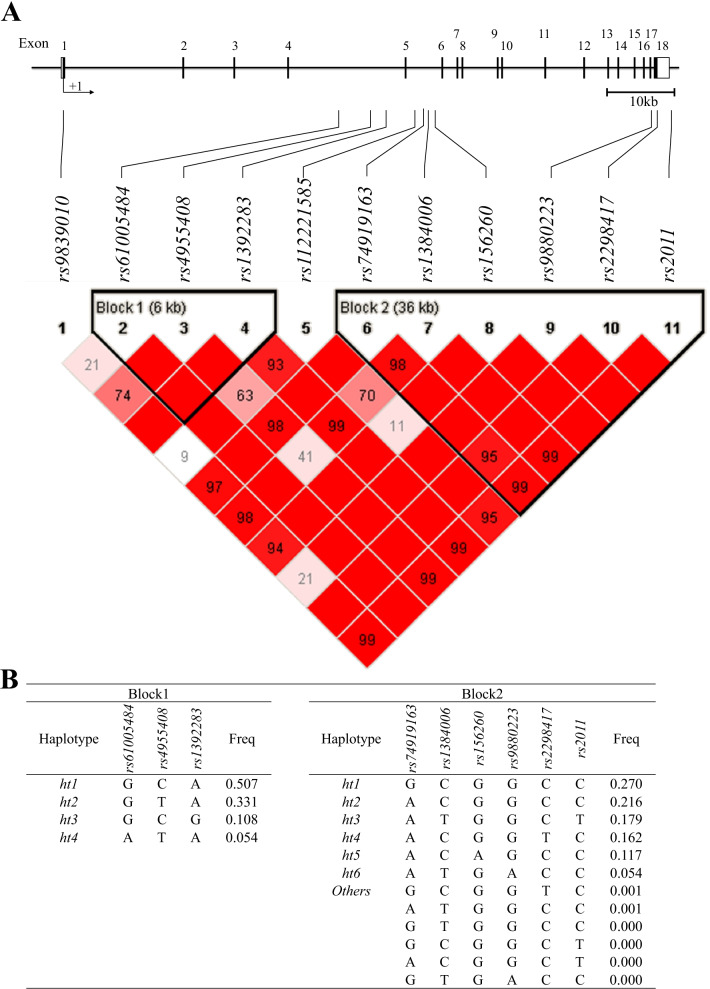
Map, SNP location and linkage disequilibrium (**A**) and haplotypes of each HapBlock (**B**) of the eleven SNPs in OXSR1 gene

Genotype frequencies of the 11 SNPs are demonstrated in Additional file 1: Table S2 and their HWEs were > 0.05. Two haplotype blocks were generated on the basis of LD among the 11 SNPs (Fig. 1A). HapBlock 1 included four haplotypes (frequency > 0.05), and HapBlock 2 included six haplotypes (Fig. 1B). Haplotypes (*ht*) *3*, and *ht4* in block 1 and *ht1, ht3, ht4, ht5,* and *ht6* in block 2 were excluded from further statistical analysis because of their equivalences to *rs1392283*, *rs61005484*, *rs74919163, rs2011, rs2298417, rs156260* and *rs9880223*, respectively.

### Association of SNPs and haplotypes on OXSR1 with annual number of exacerbations during the first year

In the total study subjects, patients with the common allele of *rs1392283* A > G showed a significantly higher annual number of exacerbations (0.43 ± 0.03 vs. 0.32 ± 0.04 vs. 0.14 ± 0.08 in dominant models, *P* = 0.043, Additional file 1: Table S3). Conversely, patients with minor alleles of *rs2298417* C > T showed a higher annual number of exacerbations (0.38 ± 0.02 vs. 0.47 ± 0.05 vs. 0.5 ± 0.12 in dominant models, *P* = 0.020, Additional file 1: Table S3). However, these differences disappeared after correction for multiple comparisons.

In non-smokers, we performed analyses on each group of never-smokers (NS = 955) and current and ex-smokers (n = 499), because smoking itself is a confounding variable affecting asthma exacerbations [34]. Clinical parameters of the two groups are presented in Additional file 1: Table S4. In the analysis using the univariate general linear model, *rs9839010, rs1392283, rs74919163, rs1384006, rs2011*, *ht1c, ht4c*, and *ht2* in block1 showed differences (*P* < 0.05) in the annual number of exacerbations depending on the genotypes in the non-smoker group (Table 2). Even after correction for multiple comparisons, patients with the common allele of *rs1384006* C > T persistently showed higher annual exacerbation numbers (0.43 ± 0.04 vs. 0.28 ± 0.03 vs. 0.31 ± 0.09 in the dominant mode, *P*corr = 0.039, Table 2). In the smoker group, there was no significant association between SNP variants and number of asthma exacerbations (Additional file 1: Table S5).

**Table 2:** Association of SNPs and haplotypes of *OXSR1* with the number of exacerbations during the 1st year of follow up in non-smoker subjects

Locus	No. of exacerbations, Mean ± SE (N)			Codomiant		Dominant		Recessive	
	CC	CR	RR	P*	*P*corr	P*	*P*corr	P*	*P*corr
*rs9839010*	0.41 ± 0.03 (600)	0.28 ± 0.03 (308)	0.34 ± 0.11 (47)	*0.038*	0.383	*0.011*	0.114	0.268	1.000
*rs61005484*	0.38 ± 0.03 (845)	0.25 ± 0.06 (108)	0.5 ± 0.5 (2)	0.445	1.000	0.218	1.000	0.881	1.000
*rs4955408*	0.31 ± 0.04 (342)	0.44 ± 0.04 (462)	0.28 ± 0.05 (150)	0.081	0.811	0.090	0.905	0.411	1.000
*rs1392283*	0.39 ± 0.03 (758)	0.27 ± 0.04 (183)	0.15 ± 0.1 (13)	*0.045*	0.453	*0.013*	0.128	0.597	1.000
*rs112221585*	0.37 ± 0.03 (817)	0.33 ± 0.07 (131)	0.33 ± 0.33 (3)	0.846	1.000	0.566	1.000	0.875	1.000
*rs74919163*	0.31 ± 0.03 (481)	0.46 ± 0.04 (389)	0.21 ± 0.07 (66)	*0.041*	0.413	*0.031*	0.307	0.532	1.000
*rs1384006*	0.43 ± 0.04 (541)	0.28 ± 0.03 (356)	0.31 ± 0.09 (58)	*0.015*	0.153	*0.004*	*0.039*	0.476	1.000
*rs156260*	0.37 ± 0.03 (736)	0.36 ± 0.06 (206)	0 ± 0 (13)	0.635	1.000	0.928	1.000	0.344	1.000
*rs9880223*	0.38 ± 0.03 (845)	0.25 ± 0.06 (108)	0.5 ± 0.5 (2)	0.445	1.000	0.218	1.000	0.881	1.000
*rs2298417*	0.35 ± 0.03 (683)	0.4 ± 0.05 (247)	0.58 ± 0.15 (24)	0.120	1.000	0.131	1.000	0.080	0.802
*rs2011*	0.4 ± 0.03 (634)	0.3 ± 0.04 (284)	0.24 ± 0.09 (37)	0.067	0.673	*0.020*	0.201	0.490	1.000
*Block1_ht1*	0.34 ± 0.05 (216)	0.42 ± 0.04 (506)	0.28 ± 0.04 (233)	0.120	0.360	0.077	0.231	0.646	1.000
*Block1_ht2*	0.3 ± 0.06 (108)	0.44 ± 0.04 (435)	0.3 ± 0.03 (412)	*0.029*	0.087	*0.025*	0.075	0.513	1.000
*Block2_ht2*	0.47 ± 0.14 (34)	0.37 ± 0.05 (309)	0.36 ± 0.03 (612)	0.768	1.000	0.814	1.000	0.469	1.000

The italic, underlined *P* values indicate statistical significance (*P* < 0.05)

*CC* common allele homozygote, *CR* heterozygote, *RR* minor allele homozygote, *SE* standard error of mean, *Pcorr* corrected *P* value for multiple comparisons

*Pcorr* corrected *P* values using the effective number of independent marker loci (M_effLi_) calculated by SNPSpD for each SNP (M_effLi_ = 10.03501), and using the number of haplotypes (n = 3) for each haplotypes

*Adjusted for age, sex, serum total IgE level, predicted FEV1% at the first visit, and total ICS and systemic steroid dose in the 1st year of visit as covariates

### Association of SNPs and haplotypes of OXSR1 with risk of frequent exacerbation

**Fig. 2:**
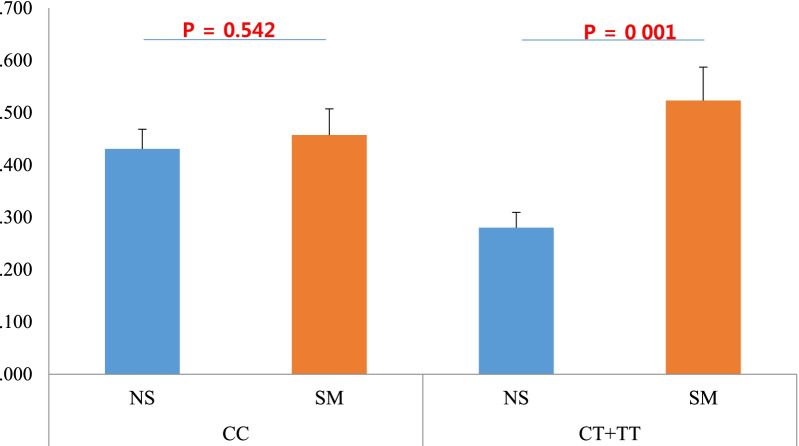
Comparison of annual number of exacerbations according to the genotypes of *rs1384006* on *OXSR1*

SNPs associated with the risk of frequent exacerbations were analyzed by logistic regression (Additional file 1: Table S6). In the non-smoker group, the common allele homozygotes of *rs1384006* C > T had significantly increased numbers of frequent exacerbators (CC vs. CR vs. RR: 74.3% vs. 20.3% vs. 5.4%, *P* = 0.004, OR: 0.36 [0.18–0.72], Fig. 2 and Table 3). This risk was significant even after correction for multiple comparisons (*P*corr: 0.038, Table 3). However, this SNP was not associated with the risk of frequent exacerbator in smokers (OR:0.93 [0.48–1.79], *P* = 0.825). In the analysis of all patients, common allele variants of *rs1392283, rs1384006*, and *rs2011* were found to be associated with an increased risk of frequent exacerbators. However, these differences lost their significance after correction for multiple comparisons (Additional file 1: Table S6).

**Table 3:** Risk of a frequent exacerbator with the SNPs and haplotypes of *OXSR1* in the non-smoker group

Locus	Exacerbation		Genotype (N, %)				Codominant			Dominant			Recessive	
		CC	CR	RR	Total	OR	*P*	*P*corr	OR	*P*	*P*corr	OR	*P*	*P*corr
*rs9839010*	Exa < 2	544 (61.7%)	293 (33.3%)	44 (5%)	881 (100%)	0.48 [0–0.86]	0.013	0.131	0.41 [0.2–0.84]	0.015	0.151	0.29 [0.06–1.47]	0.136	1.000
	Exa ≥ 2	56 (75.7%)	15 (20.3%)	3 (4.1%)	74 (100%)									
*rs61005484*	Exa < 2	778 (88.3%)	101 (11.5%)	2 (0.2%)	881 (100%)	0.86 [0.32–2.27]	0.759	1.000	0.87 [0.32–2.33]	0.779	1.000	0 [0–0]	0.999	1.000
	Exa ≥ 2	67 (90.5%)	7 (9.5%)	0 (0%)	74 (100%)									
*rs4955408*	Exa < 2	319 (36.3%)	420 (47.7%)	141 (16%)	880 (100%)	1.01 [0.65–1.59]	0.957	1.000	1.12 [0.59–2.13]	0.720	1.000	0.84 [0.34–2.05]	0.702	1.000
	Exa ≥ 2	23 (31.1%)	42 (56.8%)	9 (12.2%)	74 (100%)									
*rs1392283*	Exa < 2	693 (78.8%)	175 (19.9%)	12 (1.4%)	880 (100%)	0.53 [0.23–1.23]	0.140	1.000	0.43 [0.17–1.1]	0.078	0.784	2.03 [0.2–20.44]	0.547	1.000
	Exa ≥ 2	65 (87.8%)	8 (10.8%)	1 (1.4%)	74 (100%)									
*rs112221585*	Exa < 2	755 (86.1%)	119 (13.6%)	3 (0.3%)	877 (100%)	1.28 [0–2.81]	0.541	1.000	1.32 [0.59–2.95]	0.506	1.000	0 [0–0]	0.999	1.000
	Exa ≥ 2	62 (83.8%)	12 (16.2%)	0 (0%)	74 (100%)									
*rs74919163*	Exa < 2	450 (52.1%)	351 (40.6%)	63 (7.3%)	864 (100%)	1.22 [0–2.05]	0.461	1.000	1.29 [0.7–2.37]	0.422	1.000	1.09 [0.24–4.93]	0.912	1.000
	Exa ≥ 2	31 (43.1%)	38 (52.8%)	3 (4.2%)	72 (100%)									
*rs1384006*	Exa < 2	486 (55.2%)	341 (38.7%)	54 (6.1%)	881 (100%)	0.47 [0–0.83]	0.009	0.094	0.36 [0.18–0.72]	0.004	0.038	0.58 [0.15–2.26]	0.435	1.000
	Exa ≥ 2	55 (74.3%)	15 (20.3%)	4 (5.4%)	74 (100%)									
*rs156260*	Exa < 2	681 (77.3%)	187 (21.2%)	13 (1.5%)	881 (100%)	1.3 [0.69–2.43]	0.413	1.000	1.51 [0.77–2.98]	0.229	1.000	0 [0–0]	0.999	1.000
	Exa ≥ 2	55 (74.3%)	19 (25.7%)	0 (0%)	74 (100%)									
*rs9880223*	Exa < 2	778 (88.3%)	101 (11.5%)	2 (0.2%)	881 (100%)	0.86 [0.32–2.27]	0.759	1.000	0.87 [0.32–2.33]	0.779	1.000	0 [0–0]	0.999	1.000
	Exa ≥ 2	67 (90.5%)	7 (9.5%)	0 (0%)	74 (100%)									
*rs2298417*	Exa < 2	636 (72.3%)	225 (25.6%)	19 (2.2%)	880 (100%)	1.87 [1.13–3.09]	0.015	0.155	1.96 [1.05–3.66]	0.035	0.353	3.41 [0.97–12.01]	0.056	0.560
	Exa ≥ 2	47 (63.5%)	22 (29.7%)	5 (6.8%)	74 (100%)									
*rs2011*	Exa < 2	575 (65.3%)	270 (30.6%)	36 (4.1%)	881 (100%)	0.43 [0.22–0.84]	0.013	0.131	0.38 [0.17–0.81]	0.013	0.132	0.24 [0.03–2.05]	0.192	1.000
	Exa ≥ 2	59 (79.7%)	14 (18.9%)	1 (1.4%)	74 (100%)									
	Exa ≥ 2	0 (0%)	9 (12.2%)	65 (87.8%)	74 (100%)									
*Block1_ht1*	Exa < 2	199 (22.6%)	462 (52.4%)	220 (25%)	881 (100%)	0.82 [0.53–1.28]	0.390	1.000	0.69 [0.32–1.49]	0.347	1.000	0.85 [0.43–1.72]	0.660	1.000
	Exa ≥ 2	17 (23%)	44 (59.5%)	13 (17.6%)	74 (100%)									
*Block1_ht2*	Exa < 2	101 (11.5%)	396 (44.9%)	384 (43.6%)	881 (100%)	0.95 [0.6–1.51]	0.837	1.000	0.86 [0.46–1.58]	0.620	1.000	1.21 [0.43–3.38]	0.718	1.000
	Exa ≥ 2	7 (9.5%)	39 (52.7%)	28 (37.8%)	74 (100%)									
	Exa ≥ 2	3 (4.1%)	40 (54.1%)	31 (41.9%)	74 (100%)									
*Block2_ht2*	Exa < 2	32 (3.6%)	285 (32.3%)	564 (64%)	881 (100%)	1.34 [0.78–2.3]	0.297	0.891	1.41 [0.75–2.66]	0.289	0.867	1.42 [0.3–6.85]	0.661	1.000
	Exa ≥ 2	2 (2.7%)	24 (32.4%)	48 (64.9%)	74 (100%)									

*CC* common allele homozygote, *CR* heterozygote, *RR* minor allele homozygote, *SE* standard error of mean, *Pcorr* corrected *P* value for multiple comparisons

*Pcorr* corrected *P* values using the effective number of independent marker loci (M_effLi_) calculated by SNPSpD for each SNP (M_effLi_ = 10.03501), and using the number of haplotypes (n = 3) for each haplotypes

*Adjusted for age, sex, serum total IgE level, predicted FEV1% at the first visit, and total ICS and systemic steroid dose in the 1st year of visit as covariates

## Discussion

In this study, we were the first to demonstrate that the common allele homozygotes of *rs1384006 C* > *T* of the *OXSR1* gene were significantly associated with a higher exacerbation rate and the risk of FE in the nonsmoking asthmatics. *OXSR1* has rarely been studied with regard to respiratory diseases, although it plays a role as a salt transportation, and cell volume control through ionic mechanisms [35, 36] and ion transport by bronchial epithelial cells is essential for healthy airways. Imbalance of the transport system is closely related to the pathophysiology of asthma such as dysfunction of epithelial cells and smooth muscles [37, 38].

To the best of our knowledge, this is the first study to suggest that OXSR1 may play a role in asthma exacerbation under interaction with smoking conditions. According to Reducing Associations by Linking Genes And omics Results (REALGAR, https://realgar.org/) database [39, 40], a tissue-specific, disease-focused resource for integrating omics results, the expression of the OXSR1 gene in various cells was not only affected by smoking, but also by treatment of glucocorticoid (Additional file 2: Fig. S1). In cell-based transcriptome studies, the expression of OXSR1 was significantly increased in the bronchial epithelial cell by cigarette and e-cig smoking (effect size-based meta-FDR q value = 4.54 × 10^–8^, Additional file 2: Fig. S1A), as well as in airway smooth muscle and BEAS-2B cells by dexamethasone and budesonide treatment (meta-FDR q value = 0.032, Additional file 2: Fig. S1B). However, there was no significant association with the risk of mild to moderate asthma, severe asthma, and fetal asthma (meta-FDR q = 0.371, Additional file 2: Fig. S2). These transcriptome-based search results indicate that the OXSR1 gene is associated with smoking and glucocorticoid treatment responses, which may affect asthma exacerbation, and can provide biological bases for our observations, where the genetic association of OXSR1 with asthma exacerbation was limited to non-smokers.

Epithelial cells serve to protect airways from inhaled toxic substances and microorganisms. Airway secretory cells secrete mucin as the core glycoproteins of mucus, and cilia on the top of ciliated cells export mucus outside the lung to protect the lung from particles and pathogens [41]. This mucociliary clearance is an important innate defense mechanism that cleans up inhaled allergens and other harmful stimuli [42]. The mucus gel is placed on a fluid layer called an airway-surface liquid (ASL), and the efficacy of mucociliary clearance depends on the ion transport pathways to maintain the depth of ASL [37, 41].

Previous studies found that asthma is associated with reduced mucociliary clearance, especially during exacerbation [38, 42]. In β-epithelial Na^+^ channel (*Scnnlb*) transgenic mice, mucociliary clearance is reduced due to dehydration and thickened mucus [43]. In addition, the *Scnnlb* transgenic juvenile mice exhibit type 2 airway inflammation such as IL-13, airway eosinophilia, and alternative macrophage activation with reduced mucociliary clearance [44, 45]. In chronic lung diseases including asthma, epithelial Na^+^ channel blockers, amiloride, or hypertonic saline can restore mucociliary clearance by improving hydration of the airway surfaces. These facts support that there is a close association between the Na^+^ channel, mucociliary clearance, and asthma [46, 47]. Therefore, the *OXSR1* gene, which plays a role in regulating salt, water and cell volume by an ionic mechanism, is likely to play an important role in the mucus concentration, ASL fluid layer, and mucociliary clearance, suggesting that genetic variants of *OXSR1* are presumed to be related to frequent exacerbation of asthma through these mechanism.

Recently, oxidative stress and its pathways have been thought to contribute significantly to severe asthma and asthma exacerbations [48, 49]. However, the relationship between *OXSR1*, a gene related to oxidative stress, and asthma exacerbation has never been studied. *OXSR1* is also involved in the regulation of immune responses by interacting with TNF receptor protein kinase C-θ (PKCθ), which is expressed by lymphoid tissues [26, 36]. OXSR1 and WNK1 kinase, an upstream activator of OXSR1, are hardly detectable at basal activity, which may mean that WNK-OXSR1 signaling is regulated tightly in normal physiological conditions [36].

Interestingly, this genetic effect of *rs1384006 C* > *T* was not found in the smoker asthmatics. This indicates that there was an interaction effect between *rs1384006* and smoking status, which was confirmed by including the SNP × smoking interaction term when analyzing genetic association for total subjects. The interaction terms were statistically significant in both general linear model (F = 5.42, *P* = 0.020 (in GLM) and logistic regression analysis (OR = 2.63 [95% CI 1.02–6.83], *P* = 0.046). The reason for this finding could be explained by smoking itself being a strong inducer to exacerbate asthma [34]. Cigarette smoking is associated with accelerated decline of lung function in asthmatics [50], resulting in worsening of asthma severity [51], reduction of responsiveness to glucocorticoids [52], poor asthma control, and a higher hospital admissions [53].

The most important mechanism that may explain the relative corticosteroid resistance in smokers with asthma and COPD is a reduction in the expression of the enzyme histone deacetylase 2 (HDAC2). A reduction in HDAC activity and HDAC2 expression may account for the amplified inflammation and resistance to the actions of corticosteroids. The p38 mitogen-activated protein kinase (MAPK) pathway is also thought to play a role in corticosteroid insensitivity [54]. Thus, *rs1384006 C* > *T* of *OXSR1* might not exert any genetic effect in the enhanced MAPK- and reduced HDAC-induced airway inflammation in smokers.

There are some limitations to this study. First, the authors included current smokers as study subjects, which may raise the question of whether smoking asthma patients are truly pure asthmatics. However, 25% of asthma patients are still current smokers in the real world and smoking and asthma are very closely related and affect each other [55]. Pathophysiologically, smoking affects inflammatory condition of asthma patients, which also affects the responsiveness of treatments such as glucocorticoid [55, 56]. Therefore, smoking in asthma has long been recognized as an important factor that must be considered in reality. Second, there is still little information about the function of the *OXSR1* gene in asthma. According to functional estimation of the SNPs linked with *rs1384006* in Asian populations (SNPinfo Web Server, https://snpinfo.niehs.nih.gov/), *rs1384006* did not affect transcription factor binding, splicing sites, splicing regulation, or miRNA molecular functions. We also estimated the functional role of *rs1384006* using QTLbase (http://mulinlab.org/qtlbase). The search showed that *rs1384006* was associated with the mRNA expression of *OXSR1* in blood CD14 + monocyte, CD4 + native T cell, and neutrophil (FDR q < 0.01); C allele of *rs1384006* seemed to be associated with higher expression of *OXSR1* compared with T allele (beta value; 0.17–0.32 for C vs -0.03 for T allele). *Rs1384006* also showed significant association with the expression levels of *XYLB* and *ACVR2B*, genes located downstream of *OXSR1*, in blood CD4 + naïve T cells (FDR q = 1.87 × 10^–7^, beta = 0.17–0.30). Additionally, *rs1384006* was likely to be associated with methylation of cg00930230 on *XYLB* gene (97.7 Kb downstream from *rs1384006*) of blood neutrophils (*P* = 0.00065, beta = -0.438) and of cg10548708 at 31 kb upstream of OXSR1 gene (85.6 kb upstream of *rs1384006*) in monocytes (*p* = 0.00023, beta value = -0.379). These QTL data suggest that the C allele *rs1384006* could be associated with high *OXSR1* expression via epigenomic changes of the region around *OXSR1*, which should be evaluated in an additional functional study. Since little is known about it, it is worthy as an original discovery to be the subject of future genetic studies about asthma exacerbations. Third, we could not confirm the causal relationship between the common allele variant of *rs1384006* in the *OXSR1* gene and asthma exacerbations in this study because we had no replication data using other independent cohort subjects. The statistical power of our main finding on *rs1384006* at a given sample size was 0.995 and it is not clear what the appropriate sample size is when studying gene-environmental interactions in terms of asthma exacerbation, but further studies on larger samples are needed to achieve more reliable results. When searching several publicly available GWASs for asthma alternatively, *rs1384006* was associated with hospital admissions due to asthma (*P* = 0.043), as well as asthma-related anxiety (*P* = 0.002), bacterial pneumonia (*P* = 0.010), and the risk of asthma (*P* = 0.044) in FinnGen data (Additional file 1: Table S7), although no associations with asthma-related phenotypes was observed in other data sets (Michigan Genomics Initiative, UKBioBank, NHGRI-EBI, and GABRIEL Consortium). This discrepancy may be possibly due to ethnic difference and particularly the lack of consideration for smoking behavior in these public data. Fourth, because normal subjects were not included in the study, we cannot compare the SNP frequency with those of non-asthmatic controls. Therefore, further functional experiments are needed to identify its pathophysiology in asthma compared to normal controls.

## Conclusions

We have newly discovered that variants of the *OXSR1* gene, which is involved in the regulation of salt and cell volume, immune response, and oxidative stress, may affect asthma exacerbation. This will provide an opportunity to highlight a new genetic mechanism related to asthma exacerbation.

## Supplementary Information

**Additional file 1.** **Table S1.**. Asthma medications except glucocorticosteroids of the study subjects. **Table S2.** Minor allele frequencies, heterozygosity, and Hardy-Weinberg equilibrium of OXSR1 gene polymorphisms. **Table S3.** Association of SNPs and haplotypes of OXSR1 with the number of exacerbations during the 1st year of follow up in the total subjects. **Table S4.** Clinical characteristics of the study subjects according to their smoking status. **Table S5.** Association of SNPs and haplotypes of OXSR1 with the number of exacerbations during the 1st year of follow-up in smoker subjects. **Table S6.** Risk of frequent exacerbators with the SNPs and haplotypes of *OXSR1*. **Table S7.** Publicly available GWASs for asthma phenotypes and *rs1384006*.

**Additional file 2.** **Supplementary Figure S1.** The expression of the OXSR1 gene in various cells by smoking (A) and glucocorticoid (B) according to cell-based transcriptome studies in REALGAR database (https://realgar.org/). **Supplementary Figure S2.** The expression of the OXSR1 gene in various subtypes of asthma according to cell-based transcriptome studies in REALGAR database (https://realgar.org/).

**Additional file 3.** Genomic raw data.

## Data Availability

Genomic raw data was submitted as an Additional file 3.

## References

[1] Herrera-Luis E, Hernandez-Pacheco N, Vijverberg SJ, Flores C, Pino-Yanes M (2019). Role of genomics in asthma exacerbations. Curr Opin Pulm Med.

[2] Park HW, Tantisira KG (2017). Genetic signatures of asthma exacerbation. Allergy Asthma Immunol Res.

[3] Kim M-A, Shin S-W, Park J-S, Uh S-T, Chang HS, Bae D-J (2017). Clinical characteristics of exacerbation-prone adult asthmatics identified by cluster analysis. Allergy Asthma Immunol Res.

[4] Park SY, Jung HW, Lee JM, Shin B, Kim HJ, Kim MH (2019). Novel Trajectories for identifying asthma phenotypes: a longitudinal study in Korean Asthma Cohort, COREA. J Allergy Clin Immunol Pract.

[5] Hunninghake GM, Soto-Quiros ME, Avila L, Su J, Murphy A, Demeo DL (2007). Polymorphisms in IL13, total IgE, eosinophilia, and asthma exacerbations in childhood. J Allergy Clin Immunol.

[6] Wenzel SE, Balzar S, Ampleford E, Hawkins GA, Busse WW, Calhoun WJ (2007). IL4R alpha mutations are associated with asthma exacerbations and mast cell/IgE expression. Am J Respir Crit Care Med.

[7] Cunningham J, Basu K, Tavendale R, Palmer CN, Smith H, Mukhopadhyay S (2011). The CHI3L1 rs4950928 polymorphism is associated with asthma-related hospital admissions in children and young adults. Ann Allergy Asthma Immunol.

[8] Bisgaard H, Bonnelykke K, Sleiman PM, Brasholt M, Chawes B, Kreiner-Moller E (2009). Chromosome 17q21 gene variants are associated with asthma and exacerbations but not atopy in early childhood. Am J Respir Crit Care Med.

[9] Kljaic-Bukvic B, Blekic M, Aberle N, Curtin JA, Hankinson J, Semic-Jusufagic A (2014). Genetic variants in endotoxin signalling pathway, domestic endotoxin exposure and asthma exacerbations. Pediatr Allergy Immunol.

[10] Peters MC, Kerr S, Dunican EM, Woodruff PG, Fajt ML, Levy BD (2019). Refractory airway type 2 inflammation in a large subgroup of asthmatic patients treated with inhaled corticosteroids. J Allergy Clin Immunol.

[11] Bhavsar P, Hew M, Khorasani N, Torrego A, Barnes PJ, Adcock I (2008). Relative corticosteroid insensitivity of alveolar macrophages in severe asthma compared with non-severe asthma. Thorax.

[12] Qu J, Li Y, Zhong W, Gao P, Hu C (2017). Recent developments in the role of reactive oxygen species in allergic asthma. J Thorac Dis.

[13] Meng TC, Fukada T, Tonks NK (2002). Reversible oxidation and inactivation of protein tyrosine phosphatases in vivo. Mol Cell.

[14] Stoker AW (2005). Protein tyrosine phosphatases and signalling. J Endocrinol.

[15] Jang MK, Kim SH, Lee KY, Kim TB, Moon KA, Park CS (2010). The tyrosine phosphatase, SHP-1, is involved in bronchial mucin production during oxidative stress. Biochem Biophys Res Commun.

[16] Kuyper LM, Pare PD, Hogg JC, Lambert RK, Ionescu D, Woods R (2003). Characterization of airway plugging in fatal asthma. Am J Med.

[17] Denlinger LC, Phillips BR, Ramratnam S, Ross K, Bhakta NR, Cardet JC (2017). Inflammatory and comorbid features of patients with severe asthma and frequent exacerbations. Am J Respir Crit Care Med.

[18] Matsui H, Grubb BR, Tarran R, Randell SH, Gatzy JT, Davis CW (1998). Evidence for periciliary liquid layer depletion, not abnormal ion composition, in the pathogenesis of cystic fibrosis airways disease. Cell.

[19] Girotti AW (1985). Mechanisms of lipid peroxidation. J Free Radic Biol Med.

[20] Kelly FJ, Mudway IS (2003). Protein oxidation at the air-lung interface. Amino Acids.

[21] Qu F, Qin XQ, Cui YR, Xiang Y, Tan YR, Liu HJ (2009). Ozone stress down-regulates the expression of cystic fibrosis transmembrane conductance regulator in human bronchial epithelial cells. Chem Biol Interact.

[22] Dorwart MR, Shcheynikov N, Wang Y, Stippec S, Muallem S (2007). SLC26A9 is a Cl(−) channel regulated by the WNK kinases. J Physiol.

[23] Yang CL, Liu X, Paliege A, Zhu X, Bachmann S, Dawson DC (2007). WNK1 and WNK4 modulate CFTR activity. Biochem Biophys Res Commun.

[24] Yang D, Li Q, So I, Huang CL, Ando H, Mizutani A (2011). IRBIT governs epithelial secretion in mice by antagonizing the WNK/SPAK kinase pathway. J Clin Invest.

[25] Alessi DR, Zhang J, Khanna A, Hochdorfer T, Shang Y, Kahle KT (2014). The WNK-SPAK/OSR1 pathway: master regulator of cation-chloride cotransporters. Sci Signal.

[26] Li Y, Hu J, Vita R, Sun B, Tabata H, Altman A (2004). SPAK kinase is a substrate and target of PKCtheta in T-cell receptor-induced AP-1 activation pathway. EMBO J.

[27] Gagnon KB, Delpire E (2012). Molecular physiology of SPAK and OSR1: two Ste20-related protein kinases regulating ion transport. Physiol Rev.

[28] Reddel HK, Taylor DR, Bateman ED, Boulet LP, Boushey HA, Busse WW (2009). An official American Thoracic Society/European Respiratory Society statement: asthma control and exacerbations: standardizing endpoints for clinical asthma trials and clinical practice. Am J Respir Crit Care Med.

[29] Uh ST, Park JS, Koo SM, Kim YK, Kim KU, Kim MA (2019). Association of genetic variants of NLRP4 with exacerbation of asthma: the effect of smoking. DNA Cell Biol.

[30] Lin CH, Yeakley JM, McDaniel TK, Shen R (2009). Medium- to high-throughput SNP genotyping using VeraCode microbeads. Methods Mol Biol.

[31] Stephens M, Smith NJ, Donnelly P (2001). A new statistical method for haplotype reconstruction from population data. Am J Hum Genet.

[32] Nyholt DR (2004). A simple correction for multiple testing for single-nucleotide polymorphisms in linkage disequilibrium with each other. Am J Hum Genet.

[33] Skol AD, Scott LJ, Abecasis GR, Boehnke M (2006). Joint analysis is more efficient than replication-based analysis for two-stage genome-wide association studies. Nat Genet.

[34] Lin J, Xing B, Tang H, Yang L, Yuan Y, Gu Y (2020). Hospitalization due to asthma exacerbation: a China Asthma Research Network (CARN) retrospective study in 29 provinces across mainland China. Allergy Asthma Immunol Res.

[35] Vitari AC, Deak M, Morrice NA, Alessi DR (2005). The WNK1 and WNK4 protein kinases that are mutated in Gordon's hypertension syndrome phosphorylate and activate SPAK and OSR1 protein kinases. Biochem J.

[36] Li C, Feng M, Shi Z, Hao Q, Song X, Wang W (2014). Structural and biochemical insights into the activation mechanisms of germinal center kinase OSR1. J Biol Chem.

[37] Astrand AB, Hemmerling M, Root J, Wingren C, Pesic J, Johansson E (2015). Linking increased airway hydration, ciliary beating, and mucociliary clearance through ENaC inhibition. Am J Physiol Lung Cell Mol Physiol.

[38] Messina MS, O'Riordan TG, Smaldone GC (1991). Changes in mucociliary clearance during acute exacerbations of asthma. Am Rev Respir Dis.

[39] Kan M, Shumyatcher M, Diwadkar A, Soliman G, Himes BE (2018). Integration of transcriptomic data identifies global and cell-specific asthma-related gene expression signatures. AMIA Annu Symp Proc.

[40] Shumyatcher M, Hong R, Levin J, Himes BE (2017). Disease-specific integration of omics data to guide functional validation of genetic associations. AMIA Annu Symp Proc.

[41] Zaidman NA, Panoskaltsis-Mortari A, O'Grady SM (2016). Differentiation of human bronchial epithelial cells: role of hydrocortisone in development of ion transport pathways involved in mucociliary clearance. Am J Physiol Cell Physiol.

[42] Wanner A, Salathe M, O'Riordan TG (1996). Mucociliary clearance in the airways. Am J Respir Crit Care Med.

[43] Fritzsching B, Hagner M, Dai L, Christochowitz S, Agrawal R, van Bodegom C (2017). Impaired mucus clearance exacerbates allergen-induced type 2 airway inflammation in juvenile mice. J Allergy Clin Immunol.

[44] Livraghi A, Grubb BR, Hudson EJ, Wilkinson KJ, Sheehan JK, Mall MA (2009). Airway and lung pathology due to mucosal surface dehydration in beta}-epithelial Na+ channel-overexpressing mice: role of TNF-{alpha and IL-4R{alpha} signaling, influence of neonatal development, and limited efficacy of glucocorticoid treatment. J Immunol.

[45] Trojanek JB, Cobos-Correa A, Diemer S, Kormann M, Schubert SC, Zhou-Suckow Z (2014). Airway mucus obstruction triggers macrophage activation and matrix metalloproteinase 12-dependent emphysema. Am J Respir Cell Mol Biol.

[46] Kohler D, App E, Schmitz-Schumann M, Wurtemberger G, Matthys H (1986). Inhalation of amiloride improves the mucociliary and the cough clearance in patients with cystic fibroses. Eur J Respir Dis Suppl.

[47] Daviskas E, Anderson SD, Gonda I, Eberl S, Meikle S, Seale JP (1996). Inhalation of hypertonic saline aerosol enhances mucociliary clearance in asthmatic and healthy subjects. Eur Respir J.

[48] Mishra V, Banga J, Silveyra P (2018). Oxidative stress and cellular pathways of asthma and inflammation: therapeutic strategies and pharmacological targets. Pharmacol Ther.

[49] Aldakheel FM, Thomas PS, Bourke JE, Matheson MC, Dharmage SC, Lowe AJ (2016). Relationships between adult asthma and oxidative stress markers and pH in exhaled breath condensate: a systematic review. Allergy.

[50] Apostol GG, Jacobs DR, Tsai AW, Crow RS, Williams OD, Townsend MC (2002). Early life factors contribute to the decrease in lung function between ages 18 and 40: the coronary artery risk development in young adults study. Am J Respir Crit Care Med.

[51] Siroux V, Pin I, Oryszczyn MP, Le Moual N, Kauffmann F (2000). Relationships of active smoking to asthma and asthma severity in the EGEA study. Epidemiological study on the genetics and environment of asthma. Eur Respir J.

[52] Chalmers GW, Macleod KJ, Little SA, Thomson LJ, McSharry CP, Thomson NC (2002). Influence of cigarette smoking on inhaled corticosteroid treatment in mild asthma. Thorax.

[53] Silverman RA, Boudreaux ED, Woodruff PG, Clark S, Camargo CA (2003). Cigarette smoking among asthmatic adults presenting to 64 emergency departments. Chest.

[54] Matsumura Y (2013). Inflammatory cellular phenotypes and molecular mechanisms of glucocorticoid resistance in patients with bronchial asthma. Antiinflamm Antiallergy Agents Med Chem.

[55] Thomson NC, Chaudhuri R, Livingston E (2004). Asthma and cigarette smoking. Eur Respir J.

[56] Flodin U, Jönsson P, Ziegler J, Axelson O (1995). An epidemiologic study of bronchial asthma and smoking. Epidemiology.

