# Supplementary material for: Association of genetic variants of oxidative stress responsive kinase 1 (OXSR1) with asthma exacerbations in non-smoking asthmatics
Source: BMC Pulm Med. 2022 Jan 4;22:3. doi: 10.1186/s12890-021-01741-x (PMC8725257; doi:10.1186/s12890-021-01741-x)
Supplement: Supplementary file 1 — Additional file 1. Table S1.. Asthma medications except glucocorticosteroids of the study subjects. Table S2. Minor allele frequencies, heterozygosity, and Hardy-Weinberg equilibrium of OXSR1 gene polymorphisms. Table S3. Association of SNPs and haplotypes of OXSR1 with the number of exacerbations during the 1st year of follow up in the total subjects. Table S4. Clinical characteristics of the study subjects according to their smoking status. Table S5. Association of SNPs and haplotypes of OXSR1 with the number of exacerbations during the 1st year of follow-up in smoker subjects. Table S6. Risk of frequent exacerbators with the SNPs and haplotypes of OXSR1. Table S7. Publicly available GWASs for asthma phenotypes and rs1384006. [file 12890_2021_1741_MOESM1_ESM.docx]

**Additional file 1:** Table S1. Asthma medications except glucocorticosteroids of the study subjects

| Medications |  | Infrequent exacerbator | Frequent exacerbator | P |
| --- | --- | --- | --- | --- |
| Antibiotics | Prescribed, % | 56.6% | 69.8% | **0.004** |
|  | Dose, g/year | 40.7 ± 5.3 | 56.2 ± 7.3 | 0.319 |
| LABA | Prescribed, % | 56.1% | 80.2% | **7.64E-08** |
|  | Dose, ug/year | 432.5 ± 15.4 | 492.9 ± 38.2 | 0.171 |
| LAMA | Prescribed, % | 2.3% | 6.3% | **0.008** |
|  | Dose, ug/year | 82.3 ± 15.1 | 69.8 ± 20.8 | 0.695 |
| LTRA | Prescribed, % | 19.7% | 50.8% | **1.34E-15** |
|  | Dose, g/year | 8.2 ± 1.5 | 14.2 ± 4.1 | 0.165 |
| Mucolytics | Prescribed, % | 9.4% | 9.5% | 0.967 |
|  | Dose, g/year | 13.2 ± 1.4 | 28.7 ± 7.5 | 0.065 |
| SABA | Prescribed, % | 30.0% | 42.1% | **0.006** |
|  | Dose, mg/year | 57.7 ± 5 | 193.1 ± 94.5 | 0.159 |
| SAMA | Prescribed, % | 6.4% | 18.3% | **1.89E-05** |
|  | Dose, mg/year | 33.4 ± 3.7 | 26.9 ± 6.1 | 0.403 |
| Xanthine derivatives | Prescribed, % | 23.4% | 62.7% | **7.26E-19** |
|  | Dose, g/year | 192.3 ± 12.5 | 241.8 ± 28 | 0.110 |

LABA, long-acting beta-2 agonists; LAMA, long-acting muscarinic antagonists; LTRA, leukotriene receptor antagonist; SABA, short-acting beta-2 agonists; SAMA, short-acting muscarinic antagonists

Table S2. Minor allele frequencies, heterozygosity, and Hardy-Weinberg equilibrium of *OXSR1* gene polymorphisms

| rs number | Allele | Position | Genotype (N) | | | | MAF | Heterozygosity | HWP |
| --- | --- | --- | --- | --- | --- | --- | --- | --- | --- |
|  |  |  | C/C | C/R | R/R | Total |  |  |  |
| *rs9839010* | *T>C* | upstream-2KB | 941 | 444 | 69 | 1454 | 0.200 | 0.320 | 0.078 |
| *rs61005484* | *G>A* | intron | 1282 | 166 | 5 | 1453 | 0.061 | 0.114 | 0.879 |
| *rs4955408* | *C>T* | intron | 534 | 707 | 211 | 1452 | 0.389 | 0.475 | 0.350 |
| *rs1392283* | *A>G* | intron | 1168 | 264 | 21 | 1453 | 0.105 | 0.188 | 0.173 |
| *rs112221585* | *T>G* | intron | 1233 | 202 | 9 | 1444 | 0.076 | 0.141 | 0.817 |
| *rs74919163* | *A>G* | intron | 748 | 576 | 98 | 1422 | 0.271 | 0.396 | 0.363 |
| *rs1384006* | *C>T* | intron | 845 | 523 | 86 | 1454 | 0.239 | 0.364 | 0.671 |
| *rs156260* | *G>A* | intron | 1123 | 307 | 23 | 1453 | 0.121 | 0.213 | 0.701 |
| *rs9880223* | *G>A* | intron | 1280 | 166 | 5 | 1451 | 0.061 | 0.114 | 0.877 |
| *rs2298417* | *C>T* | utr-3-prime | 1017 | 390 | 46 | 1453 | 0.166 | 0.277 | 0.253 |
| *rs2011* | *C>T* | utr-3-prime | 991 | 409 | 54 | 1454 | 0.178 | 0.292 | 0.149 |

C, common allele; R, minor allele; MAF, minor allele frequency; HWP, Hardy-Weinberg P value

Table S3. Association of SNPs and haplotypes of *OXSR1* with the number of exacerbations during the 1^st^ year of follow up in the total subjects

| Locus | No. of exacerbation, Mean ± SE (N) | | |  | Codomiant | |  | Dominant | |  | Recessive | |
| --- | --- | --- | --- | --- | --- | --- | --- | --- | --- | --- | --- | --- |
|  | CC | CR | RR |  | P* | Pcorr |  | P* | Pcorr |  | P* | Pcorr |
| *rs9839010* | 0.43 ± 0.03 (941) | 0.36 ± 0.03 (444) | 0.35 ± 0.08 (69) |  | 0.246 | 1.000 |  | 0.230 | 1.000 |  | 0.140 | 1.000 |
| *rs61005484* | 0.41 ± 0.02 (1282) | 0.37 ± 0.06 (166) | 0.4 ± 0.24 (5) |  | 0.569 | 1.000 |  | 0.302 | 1.000 |  | 0.947 | 1.000 |
| *rs4955408* | 0.37 ± 0.03 (534) | 0.44 ± 0.03 (707) | 0.39 ± 0.05 (211) |  | 0.583 | 1.000 |  | 0.522 | 1.000 |  | 0.571 | 1.000 |
| *rs1392283* | 0.43 ± 0.03 (1168) | 0.32 ± 0.04 (264) | 0.14 ± 0.08 (21) |  | 0.121 | 1.000 |  | *0.043* | 0.427 |  | 0.414 | 1.000 |
| *rs112221585* | 0.41 ± 0.02 (1233) | 0.39 ± 0.06 (202) | 0.56 ± 0.24 (9) |  | 0.633 | 1.000 |  | 0.437 | 1.000 |  | 0.692 | 1.000 |
| *rs74919163* | 0.37 ± 0.03 (748) | 0.45 ± 0.04 (576) | 0.31 ± 0.07 (98) |  | 0.174 | 1.000 |  | 0.286 | 1.000 |  | 0.247 | 1.000 |
| *rs1384006* | 0.44 ± 0.03 (845) | 0.37 ± 0.03 (523) | 0.31 ± 0.07 (86) |  | 0.201 | 1.000 |  | 0.087 | 0.875 |  | 0.311 | 1.000 |
| *rs156260* | 0.41 ± 0.02 (1123) | 0.4 ± 0.05 (307) | 0.13 ± 0.07 (23) |  | 0.440 | 1.000 |  | 0.832 | 1.000 |  | 0.239 | 1.000 |
| *rs9880223* | 0.41 ± 0.02 (1280) | 0.37 ± 0.06 (166) | 0.4 ± 0.24 (5) |  | 0.565 | 1.000 |  | 0.299 | 1.000 |  | 0.948 | 1.000 |
| *rs2298417* | 0.38 ± 0.02 (1017) | 0.47 ± 0.05 (390) | 0.5 ± 0.12 (46) |  | 0.066 | 0.663 |  | *0.020* | 0.199 |  | 0.451 | 1.000 |
| *rs2011* | 0.43 ± 0.03 (991) | 0.37 ± 0.04 (409) | 0.24 ± 0.07 (54) |  | 0.353 | 1.000 |  | 0.231 | 1.000 |  | 0.263 | 1.000 |
| *Block1_ht1* | 0.4 ± 0.04 (352) | 0.43 ± 0.03 (768) | 0.35 ± 0.04 (334) |  | 0.259 | 1.000 |  | 0.104 | 0.416 |  | 0.777 | 1.000 |
| *Block1_ht2* | 0.39 ± 0.07 (153) | 0.45 ± 0.03 (648) | 0.37 ± 0.03 (653) |  | 0.313 | 1.000 |  | 0.234 | 0.934 |  | 0.600 | 1.000 |
| *Block2_ht2* | 0.5 ± 0.11 (56) | 0.39 ± 0.04 (473) | 0.41 ± 0.03 (925) |  | 0.791 | 1.000 |  | 0.498 | 1.000 |  | 0.783 | 1.000 |
|  |  |  |  |  |  |  |  |  |  |  |  |  |
| *adjusted for age, sex, serum total IgE level, predicted FEV1% at the first visit, and total ICS and systemic steroid dose in the 1st year of visit as covariates. | | | | | | | | | | | |  |
| CC, common allele homozygote; CR, heterozygote; RR, minor allele homozygote; SE, standard error of mean; Pcorr, corrected P value for multiple comparisons | | | | | | | | | | | | |
| Pcorr; Corrected P values for multiple comparisons using the effective number of independent marker loci (M_effLi_) calculated by SNPSpD for each SNP (M_effLi_ = 10.03501), and using the number of haplotypes (n = 3) for each haplotype. | | | | | | | | | | | | |

Table S4. Clinical characteristics of the study subjects according to their smoking status

|  | Never-smokers | Smokers including ex-smokers | P |
| --- | --- | --- | --- |
| Number | 955 | 499 | - |
| Age (years) | 45.94 ± 0.5 | 48.14 ± 0.69 | 0.010 |
| Sex (male %) | 15.71% | 81.76% | 1.57E-133 |
| Number of exacerbations in the first year | 0.37 ± 0.02 | 0.48 ± 0.04 | 0.012 |
| Smoking status (NS/ES/SM, %) | 100%/0%/0% | 0%/54.9%/45.1% | 0.00E+00 |
| Smoking amount (pack-year) | 0 ± 0 | 17.73 ± 0.82 | 4.53E-74 |
| Atopy (%) | 47.96% | 48.10% | 0.960 |
| Duration of asthma (years) | 3.36 ± 0.25 | 3.21 ± 0.31 | 0.717 |
| Duration of follow-up (years) | 6.3 ± 0.15 | 6.21 ± 0.19 | 0.694 |
| Serum total IgE (IU/ml) | 296.68 ± 19.1 | 494.85 ± 37.21 | 2.58E-06 |
| Body mass index (kg/m^2^) | 23.7 ± 0.14 | 24.23 ± 0.2 | 0.028 |
| Baseline FVC%, predicted | 83.04 ± 0.57 | 79.1 ± 0.83 | 7.17E-05 |
| Baseline FEV1%, predicted | 83.57 ± 0.69 | 74.59 ± 1.05 | 1.86E-12 |
| Baseline FEV1/FVC | 77 ± 0.38 | 70.31 ± 0.62 | 2.45E-19 |
| PC20, methacholine (mg/ml) (No. of study subjects) | 7.88 ± 0.31 (891) | 6.51 ± 0.54 (436) | 0.019 |
| Total ICS dosage used in the 1st year (Fluticasone eqv./day) | 223.77 ± 9.57 | 278.52 ± 14.67 | 0.002 |
| Systemic prednisolone dose in the 1st year (mg/year) | 117.97 ± 15.17 | 130.39 ± 22.09 | 0.638 |

Table S5. Association of SNPs and haplotypes of *OXSR1* with the number of exacerbations during the 1^st^ year of follow-up in smoker subjects

| Locus | No. of exacerbation, Mean ± SE (N) | | |  | Codomiant | |  | Dominant | |  | Recessive | |
| --- | --- | --- | --- | --- | --- | --- | --- | --- | --- | --- | --- | --- |
|  | CC | CR | RR |  | P* | Pcorr |  | P* | Pcorr |  | P* | Pcorr |
| *rs9839010* | 0.47 ± 0.05 (341) | 0.54 ± 0.08 (136) | 0.36 ± 0.12 (22) |  | 0.198 | 1.000 |  | 0.242 | 1.000 |  | 0.342 | 1.000 |
| *rs61005484* | 0.47 ± 0.04 (437) | 0.6 ± 0.12 (58) | 0.33 ± 0.33 (3) |  | 0.983 | 1.000 |  | 0.861 | 1.000 |  | 0.990 | 1.000 |
| *rs4955408* | 0.47 ± 0.06 (192) | 0.44 ± 0.05 (245) | 0.67 ± 0.14 (61) |  | 0.538 | 1.000 |  | 0.317 | 1.000 |  | 0.864 | 1.000 |
| *rs1392283* | 0.5 ± 0.05 (410) | 0.42 ± 0.08 (81) | 0.13 ± 0.13 (8) |  | 0.842 | 1.000 |  | 0.843 | 1.000 |  | 0.558 | 1.000 |
| *rs112221585* | 0.48 ± 0.04 (416) | 0.51 ± 0.1 (71) | 0.67 ± 0.33 (6) |  | 0.676 | 1.000 |  | 0.589 | 1.000 |  | 0.589 | 1.000 |
| *rs74919163* | 0.48 ± 0.05 (267) | 0.44 ± 0.06 (187) | 0.5 ± 0.17 (32) |  | 0.477 | 1.000 |  | 0.347 | 1.000 |  | 0.310 | 1.000 |
| *rs1384006* | 0.46 ± 0.05 (304) | 0.56 ± 0.07 (167) | 0.32 ± 0.1 (28) |  | 0.456 | 1.000 |  | 0.428 | 1.000 |  | 0.491 | 1.000 |
| *rs156260* | 0.49 ± 0.05 (387) | 0.48 ± 0.08 (101) | 0.3 ± 0.15 (10) |  | 0.653 | 1.000 |  | 0.663 | 1.000 |  | 0.508 | 1.000 |
| *rs9880223* | 0.47 ± 0.04 (435) | 0.6 ± 0.12 (58) | 0.33 ± 0.33 (3) |  | 0.981 | 1.000 |  | 0.851 | 1.000 |  | 0.992 | 1.000 |
| *rs2298417* | 0.45 ± 0.05 (334) | 0.58 ± 0.08 (143) | 0.41 ± 0.2 (22) |  | 0.119 | 1.000 |  | 0.088 | 0.883 |  | 0.569 | 1.000 |
| *rs2011* | 0.48 ± 0.05 (357) | 0.53 ± 0.08 (125) | 0.24 ± 0.11 (17) |  | 0.300 | 1.000 |  | 0.328 | 1.000 |  | 0.389 | 1.000 |
| *Block1_ht1* | 0.49 ± 0.08 (136) | 0.46 ± 0.05 (262) | 0.52 ± 0.09 (101) |  | 0.652 | 1.000 |  | 0.668 | 1.000 |  | 0.360 | 1.000 |
| *Block1_ht2* | 0.62 ± 0.17 (45) | 0.46 ± 0.06 (213) | 0.48 ± 0.06 (241) |  | 0.729 | 1.000 |  | 0.447 | 1.000 |  | 0.999 | 1.000 |
| *Block2_ht2* | 0.55 ± 0.16 (22) | 0.41 ± 0.07 (164) | 0.51 ± 0.05 (313) |  | 0.624 | 1.000 |  | 0.421 | 1.000 |  | 0.779 | 1.000 |
|  |  |  |  |  |  |  |  |  |  |  |  |  |
| *adjusted for age, sex, serum total IgE level, predicted FEV1% at the first visit, and total ICS and systemic steroid dose in the 1st year of visit as covariates. | | | | | | | | | | | |  |
| CC, common allele homozygote; CR, heterozygote; RR, minor allele homozygote; SE, standard error of mean; Pcorr, corrected P value for multiple comparisons | | | | | | | | | | | | |
| Pcorr; Corrected P values for multiple comparisons using the effective number of independent marker loci (M_effLi_) calculated by SNPSpD for each SNP (M_effLi_ = 10.03501), and using the number of haplotypes (n = 3) for each haplotype. | | | | | | | | | | | | |

Table S6. Risk of frequent exacerbators with the SNPs and haplotypes of *OXSR1*

| Locus | Exacerbation | Genotype (N, %) | | | |  | Codomiant | | |  | Dominant | | |  | Recessive | | |
| --- | --- | --- | --- | --- | --- | --- | --- | --- | --- | --- | --- | --- | --- | --- | --- | --- | --- |
|  |  | CC | CR | RR | Total |  | OR | P | Pcorr |  | OR | P | Pcorr |  | OR | P | Pcorr |
| *rs9839010* | Exa <2 | 850 (64%) | 413 (31.1%) | 65 (4.9%) | 1328 (100%) |  | 0.67 [0.45 - 1.01] | 0.054 | 0.537 |  | 0.69 [0.43 - 1.11] | 0.129 | 1.000 |  | 0.26 [0.06 - 1.15] | 0.076 | 0.766 |
|  | Exa ≥2 | 91 (72.2%) | 31 (24.6%) | 4 (3.2%) | 126 (100%) |  |  |  |  |  |  |  |  |  |  |  |  |
| *rs61005484* | Exa <2 | 1169 (88.1%) | 153 (11.5%) | 5 (0.4%) | 1327 (100%) |  | 0.75 [0.37 - 1.54] | 0.438 | 1.000 |  | 0.77 [0.37 - 1.59] | 0.479 | 1.000 |  | 0 [0 - 0] | 0.999 | 1.000 |
|  | Exa ≥2 | 113 (89.7%) | 13 (10.3%) | 0 (0%) | 126 (100%) |  |  |  |  |  |  |  |  |  |  |  |  |
| *rs4955408* | Exa <2 | 492 (37.1%) | 641 (48.3%) | 193 (14.6%) | 1326 (100%) |  | 0.98 [0.71 - 1.35] | 0.889 | 1.000 |  | 1.02 [0.65 - 1.61] | 0.916 | 1.000 |  | 0.87 [0.46 - 1.65] | 0.678 | 1.000 |
|  | Exa ≥2 | 42 (33.3%) | 66 (52.4%) | 18 (14.3%) | 126 (100%) |  |  |  |  |  |  |  |  |  |  |  |  |
| *rs1392283* | Exa <2 | 1056 (79.6%) | 251 (18.9%) | 20 (1.5%) | 1327 (100%) |  | 0.55 [0.3 - 1.04] | 0.064 | 0.644 |  | 0.5 [0.26 - 0.98] | 0.045 | 0.451 |  | 0.95 [0.12 - 7.77] | 0.964 | 1.000 |
|  | Exa ≥2 | 112 (88.9%) | 13 (10.3%) | 1 (0.8%) | 126 (100%) |  |  |  |  |  |  |  |  |  |  |  |  |
| *rs112221585* | Exa <2 | 1125 (85.4%) | 184 (14%) | 9 (0.7%) | 1318 (100%) |  | 0.88 [0.48 - 1.62] | 0.678 | 1.000 |  | 0.9 [0.48 - 1.7] | 0.753 | 1.000 |  | 0 [0 - 0] | 0.999 | 1.000 |
|  | Exa ≥2 | 108 (85.7%) | 18 (14.3%) | 0 (0%) | 126 (100%) |  |  |  |  |  |  |  |  |  |  |  |  |
| *rs74919163* | Exa <2 | 694 (53.3%) | 519 (39.8%) | 90 (6.9%) | 1303 (100%) |  | 1.19 [0.83 - 1.72] | 0.346 | 1.000 |  | 1.22 [0.78 - 1.91] | 0.376 | 1.000 |  | 1.28 [0.52 - 3.18] | 0.592 | 1.000 |
|  | Exa ≥2 | 54 (45.4%) | 57 (47.9%) | 8 (6.7%) | 119 (100%) |  |  |  |  |  |  |  |  |  |  |  |  |
| *rs1384006* | Exa <2 | 757 (57%) | 490 (36.9%) | 81 (6.1%) | 1328 (100%) |  | 0.6 [0.4 - 0.9] | 0.013 | 0.128 |  | 0.57 [0.36 - 0.91] | 0.017 | 0.176 |  | 0.4 [0.12 - 1.39] | 0.150 | 1.000 |
|  | Exa ≥2 | 88 (69.8%) | 33 (26.2%) | 5 (4%) | 126 (100%) |  |  |  |  |  |  |  |  |  |  |  |  |
| *rs156260* | Exa <2 | 1026 (77.3%) | 279 (21%) | 22 (1.7%) | 1327 (100%) |  | 1.13 [0.72 - 1.78] | 0.581 | 1.000 |  | 1.26 [0.76 - 2.09] | 0.362 | 1.000 |  | 0.39 [0.05 - 3.3] | 0.391 | 1.000 |
|  | Exa ≥2 | 97 (77%) | 28 (22.2%) | 1 (0.8%) | 126 (100%) |  |  |  |  |  |  |  |  |  |  |  |  |
| *rs9880223* | Exa <2 | 1167 (88.1%) | 153 (11.5%) | 5 (0.4%) | 1325 (100%) |  | 0.75 [0.37 - 1.53] | 0.435 | 1.000 |  | 0.77 [0.37 - 1.59] | 0.475 | 1.000 |  | 0 [0 - 0] | 0.999 | 1.000 |
|  | Exa ≥2 | 113 (89.7%) | 13 (10.3%) | 0 (0%) | 126 (100%) |  |  |  |  |  |  |  |  |  |  |  |  |
| *rs2298417* | Exa <2 | 938 (70.7%) | 349 (26.3%) | 40 (3%) | 1327 (100%) |  | 1.48 [1.03 - 2.12] | 0.033 | 0.327 |  | 1.69 [1.08 - 2.65] | 0.021 | 0.211 |  | 1.39 [0.52 - 3.75] | 0.514 | 1.000 |
|  | Exa ≥2 | 79 (62.7%) | 41 (32.5%) | 6 (4.8%) | 126 (100%) |  |  |  |  |  |  |  |  |  |  |  |  |
| *rs2011* | Exa <2 | 894 (67.3%) | 381 (28.7%) | 53 (4%) | 1328 (100%) |  | 0.61 [0.39 - 0.95] | 0.030 | 0.300 |  | 0.62 [0.37 - 1.03] | 0.063 | 0.635 |  | 0.18 [0.02 - 1.4] | 0.102 | 1.000 |
|  | Exa ≥2 | 97 (77%) | 28 (22.2%) | 1 (0.8%) | 126 (100%) |  |  |  |  |  |  |  |  |  |  |  |  |
| *Block1_ht1* | Exa <2 | 320 (24.1%) | 697 (52.5%) | 311 (23.4%) | 1328 (100%) |  | 0.82 [0.6 - 1.12] | 0.209 | 0.836 |  | 0.66 [0.37 - 1.16] | 0.148 | 0.593 |  | 0.86 [0.53 - 1.4] | 0.543 | 1.000 |
|  | Exa ≥2 | 32 (25.4%) | 71 (56.3%) | 23 (18.3%) | 126 (100%) |  |  |  |  |  |  |  |  |  |  |  |  |
| *Block1_ht2* | Exa <2 | 139 (10.5%) | 587 (44.2%) | 602 (45.3%) | 1328 (100%) |  | 0.96 [0.69 - 1.34] | 0.809 | 1.000 |  | 0.9 [0.58 - 1.39] | 0.628 | 1.000 |  | 1.11 [0.53 - 2.33] | 0.784 | 1.000 |
|  | Exa ≥2 | 14 (11.1%) | 61 (48.4%) | 51 (40.5%) | 126 (100%) |  |  |  |  |  |  |  |  |  |  |  |  |
| *Block2_ht2* | Exa <2 | 52 (3.9%) | 433 (32.6%) | 843 (63.5%) | 1328 (100%) |  | 1.21 [0.82 - 1.78] | 0.338 | 1.000 |  | 1.26 [0.8 - 2] | 0.318 | 1.000 |  | 1.22 [0.41 - 3.6] | 0.722 | 1.000 |
|  | Exa ≥2 | 4 (3.2%) | 40 (31.7%) | 82 (65.1%) | 126 (100%) |  |  |  |  |  |  |  |  |  |  |  |  |
|  |  |  |  |  |  |  |  |  |  |  |  |  |  |  |  |  |  |
| *adjusted for age, sex, serum total IgE level, predicted FEV1% at the first visit, and total ICS and systemic steroid dose in the 1st year of visit as covariates. | | | | | | | | | | |  |  |  |  |  |  |  |
| CC, common allele homozygote; CR, heterozygote; RR, minor allele homozygote; SE, standard error of mean; Pcorr, corrected P value for multiple comparisons | | | | | | | | | | | |  |  |  |  |  |  |
| Pcorr; Corrected P values for multiple comparisons using the effective number of independent marker loci (M_effLi_) calculated by SNPSpD for each SNP (M_effLi_ = 10.03501), and using the number of haplotypes (n = 3) for each haplotype. | | | | | | | | | | | | | | | | | |

Table S7. Publicly available GWASs for asthma phenotypes and *rs1384006*

| Data Source | Category | Phenotype | Effect Size (beta) | P-value | Number of samples (affected / unaffected) |
| --- | --- | --- | --- | --- | --- |
| FinnGen | Comorbidities of Asthma | Anxiety (asthma-related co-morbidities) | -0.047 | 0.002 | 12513 / 180852 |
|  | Asthma and related endpoints | Bacterial pneumonia (organism specified) | 0.042 | 0.010 | 8269 / 206999 |
|  | Asthma and related endpoints | Asthma, hospital admissions, main diagnosis only | -0.024 | 0.043 | 17433 / 201359 |
|  | X Diseases of the respiratory system (J10_) | Asthma (only as main-diagnosis) | -0.025 | 0.044 | 17438 / 135449 |
|  | X Diseases of the respiratory system (J10_) | Asthma (only as main-diagnosis) (more controls excluded) | -0.025 | 0.046 | 17438 / 131051 |
| MGI | respiratory | Asthma with exacerbation | - | 0.100 | 1506 / 32282 |
|  | respiratory | Chronic obstructive asthma | - | 0.300 | 353 / 32282 |
|  | respiratory | Asthma | - | 0.320 | 6079 / 32282 |
|  | respiratory | Chronic obstructive asthma with exacerbation | - | 0.870 | 66 / 32282 |
| UKBB | respiratory | Asthma | -0.013 | 0.180 | 26332 / 375505 |
|  | respiratory | Asthma with exacerbation | 0.0053 | 0.950 | 252 / 375505 |
| GABRIEL | - | Asthma | 0.988 | 0.578 | 10365 / 16110 |
