# Supplementary material for: Association of genetic variants of oxidative stress responsive kinase 1 (OXSR1) with asthma exacerbations in non-smoking asthmatics
Source: BMC Pulm Med. 2022 Jan 4;22:3. doi: 10.1186/s12890-021-01741-x (PMC8725257; doi:10.1186/s12890-021-01741-x)
Supplement: Supplementary file 2 — Additional file 2. Supplementary Figure S1. The expression of the OXSR1 gene in various cells by smoking (A) and glucocorticoid (B) according to cell-based transcriptome studies in REALGAR database (https://realgar.org/). Supplementary Figure S2. The expression of the OXSR1 gene in various subtypes of asthma according to cell-based transcriptome studies in REALGAR database (https://realgar.org/). [file 12890_2021_1741_MOESM2_ESM.pptx]

## Slide 1
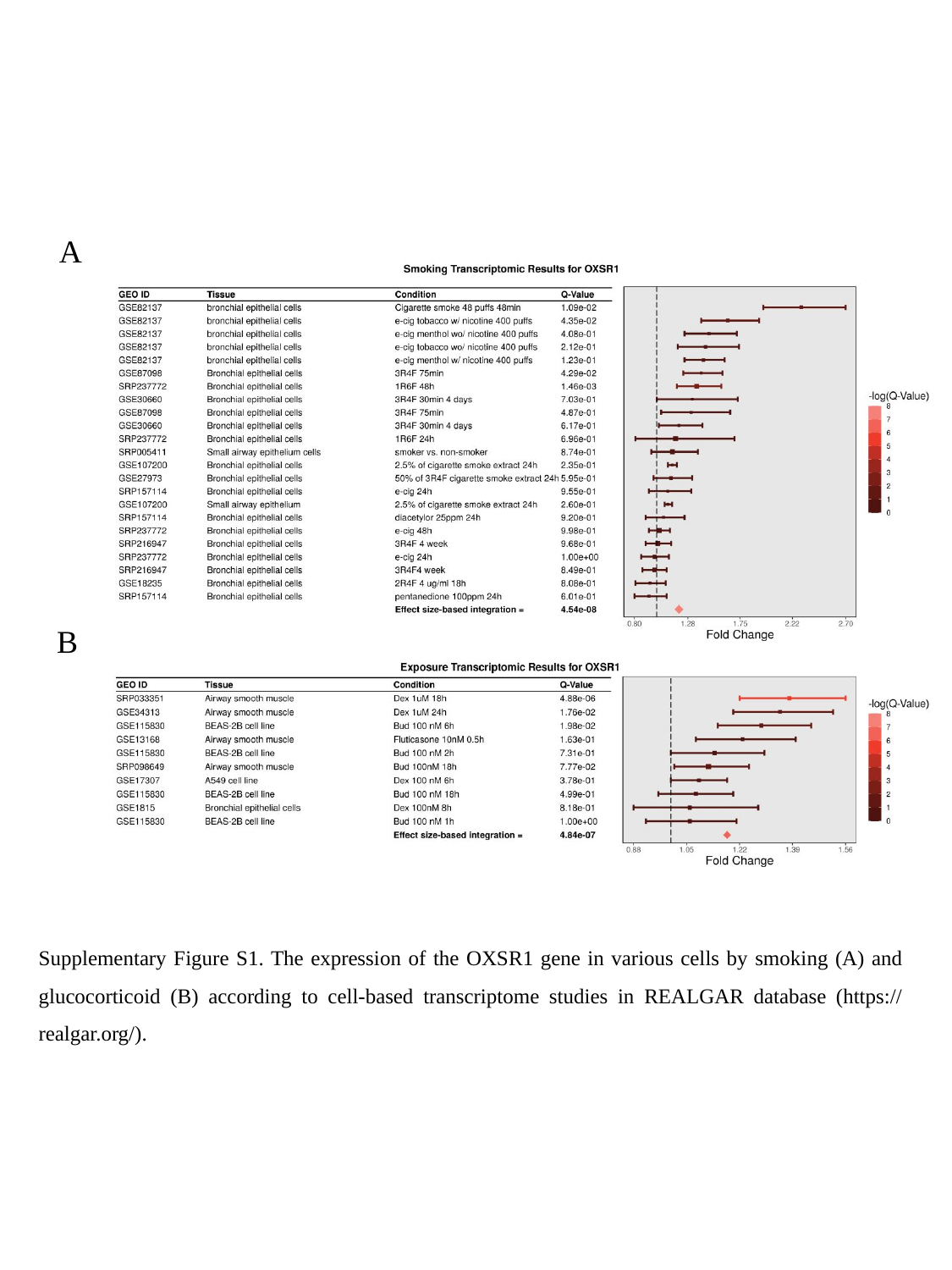

A
B
Supplementary Figure S1. The expression of the OXSR1 gene in various cells by smoking (A) and glucocorticoid (B) according to cell-based transcriptome studies in REALGAR database (https://realgar.org/).

## Slide 2
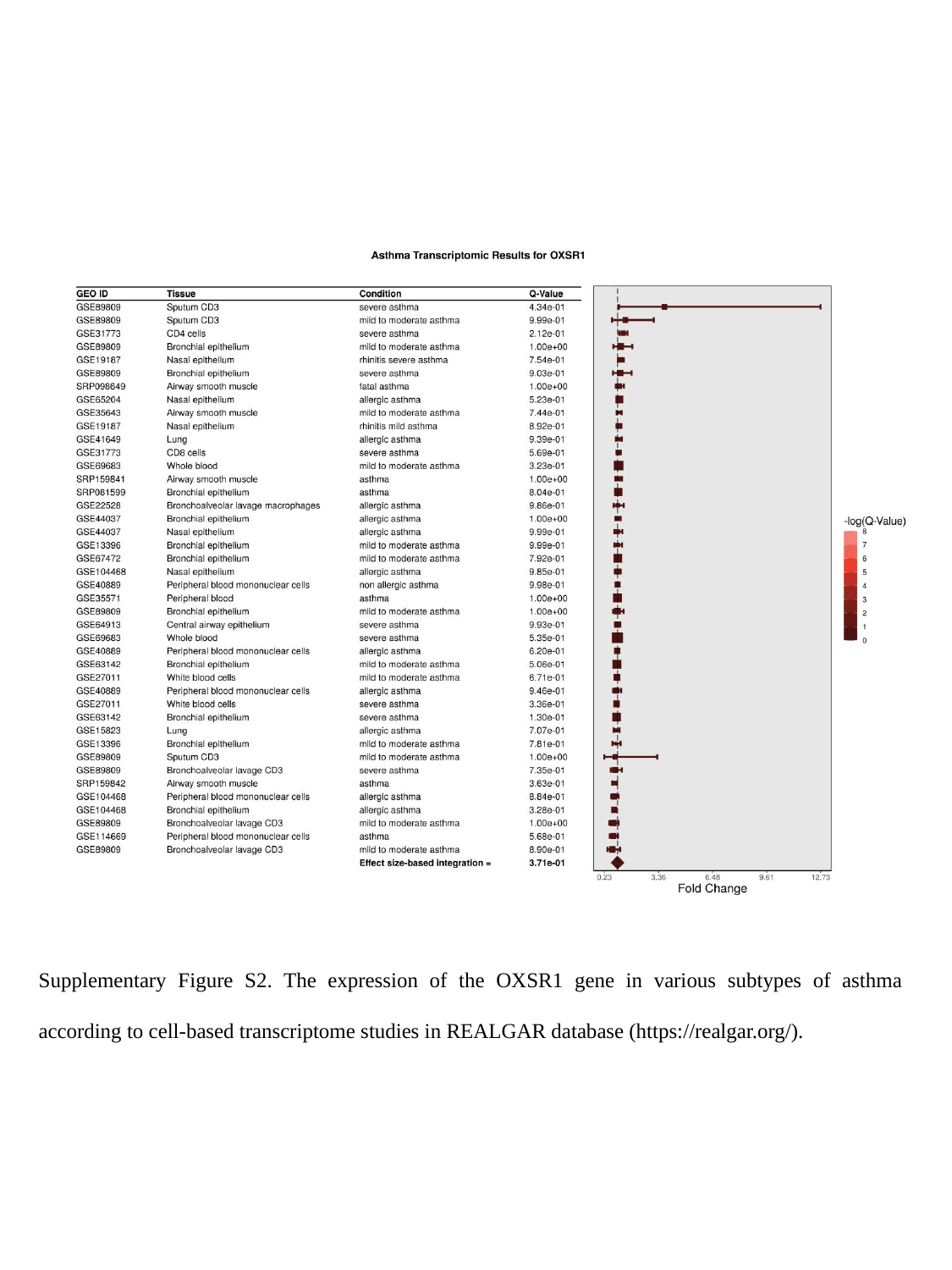

Supplementary Figure S2. The expression of the OXSR1 gene in various subtypes of asthma according to cell-based transcriptome studies in REALGAR database (https://realgar.org/).
